# Diarylureas: Repositioning from Antitumor to Antimicrobials or Multi-Target Agents against New Pandemics

**DOI:** 10.3390/antibiotics10010092

**Published:** 2021-01-19

**Authors:** Alessia Catalano, Domenico Iacopetta, Michele Pellegrino, Stefano Aquaro, Carlo Franchini, Maria Stefania Sinicropi

**Affiliations:** 1 Department of Pharmacy-Drug Sciences, University of Bari Aldo Moro, 70126 Bari, Italy; carlo.franchini@uniba.it; 2 Department of Pharmacy, Health and Nutritional Sciences, University of Calabria, 87036 Arcavacata di Rende, Italy; domenico.iacopetta@unical.it (D.I.); michele.pellegrino@unical.it (M.P.); stefano.aquaro@unical.it (S.A.); s.sinicropi@unical.it (M.S.S.)

**Keywords:** antimicrobials, antimalarial, antiviral, diarylurea, bis-arylurea, pandemics, COVID-19

## Abstract

Antimicrobials have allowed medical advancements over several decades. However, the continuous emergence of antimicrobial resistance restricts efficacy in treating infectious diseases. In this context, the drug repositioning of already known biological active compounds to antimicrobials could represent a useful strategy. In 2002 and 2003, the SARS-CoV pandemic immobilized the Far East regions. However, the drug discovery attempts to study the virus have stopped after the crisis declined. Today’s COVID-19 pandemic could probably have been avoided if those efforts against SARS-CoV had continued. Recently, a new coronavirus variant was identified in the UK. Because of this, the search for safe and potent antimicrobials and antivirals is urgent. Apart from antiviral treatment for severe cases of COVID-19, many patients with mild disease without pneumonia or moderate disease with pneumonia have received different classes of antibiotics. Diarylureas are tyrosine kinase inhibitors well known in the art as anticancer agents, which might be useful tools for a reposition as antimicrobials. The first to come onto the market as anticancer was sorafenib, followed by some other active molecules. For this interesting class of organic compounds antimicrobial, antiviral, antithrombotic, antimalarial, and anti-inflammatory properties have been reported in the literature. These numerous properties make these compounds interesting for a new possible pandemic considering that, as well as for other viral infections also for CoVID-19, a multitarget therapeutic strategy could be favorable. This review is meant to be an overview on diarylureas, focusing on their biological activities, not dwelling on the already known antitumor activity. Quite a lot of papers present in the literature underline and highlight the importance of these molecules as versatile scaffolds for the development of new and promising antimicrobials and multitarget agents against new pandemic events.

## 1. Introduction

Today, bacterial resistance represents a critical problem for human health and the care of patients with numerous diseases, thus urgently calling for the immediate development and use in therapy of drugs against multi-resistant pathogens [1]. This has encouraged the research and investigation of various drug discovery approaches: one of them is drug repurposing, which is a strategy to establish new applications for approved or investigational drugs, aside from the original medical indications. Drug repurposing is becoming more appealing because it allows for overcoming the restrictions related to the expense and delay of the discovery and development of new drugs [2,3]. In the last twenty years, humans have encountered five pandemic diseases, severe acute respiratory syndrome (SARS), Avian flu, Ebola, Middle East respiratory syndrome (MERS) [4], and coronavirus disease 2019 (COVID-19) [5,6,7]. Recently, a new variant of COVID-19 was also described [8]. It has been reported that the disease prognosis of COVID-19 is largely influenced by multi-organ involvement. The multi-organ dysfunction is characterized by acute lung and liver failure, acute kidney injury, cardiovascular disease, and a wide spectrum of hematological abnormalities and neurological disorders. The most important mechanisms are related to the direct and indirect pathogenic features induced by SARS-CoV2. Moreover, organ failure may be induced by the cytokine storm, as a result of increased levels of inflammatory mediators, endothelial dysfunction, coagulation abnormalities, and the infiltration of inflammatory cells into the organs [9,10]. Medical treatment options for COVID-19 include antiviral drugs, chloroquine/hydroxychloroquine, antibiotics, beta-blockers, steroidal and nonsteroidal anti-inflammatory drugs, and some others [11,12]. In this framework, new multitarget molecules could be useful to counteract the effects of the virus at various levels and in different ways. The repositioning of anticancer diarylureas for new indications, such as antimicrobial [13], antiviral, and anti-inflammatory, could be a very interesting option for obtaining multitarget agents useful for the treatment or prevention of pandemics. Diarylureas or bis-arylureas are generally known as anticancer agents [14], for instance sorafenib, regorafenib, linifanib, tivozanib, and ripretinib, which have been recently reviewed by our research group [15]. Nevertheless, it is reported that they have also a lot of different and important biological activities [16,17]. Indeed, they are antimicrobials, particularly active in schistosomiasis and malaria [18,19,20], anti-inflammatory, antiplatelet, and antiviral agents [21,22], and allosteric modulators of the cannabinoid CB1 receptor [23], VLA-4 integrin antagonists [24], and EGFR inhibitors [25]. Taking into account the multiple factors involved in the pathogenesis of COVID-19, all the activities of diarylureas make them promising tools for the treatment of this disease. It is known that the novel coronavirus pneumonia is closely associated with inflammatory storms. Indeed, the patients with severe COVID-19 have a “cytokine storm” syndrome, given the high-levels of pro-inflammatory cytokines found in these patients [26]. Moreover, there is an increasing recognition of a prothrombotic state in COVID-19 [27] and neuropsychiatric diseases such as depression and anxiety [28]. The expression levels of angiotensin converting enzyme 2 (ACE2), acetylcholinesterase (AChE), interleukin-6 (IL-6), based on their involvement in both environmental responses and comorbid conditions, such as hypertension and type 2 diabetes mellitus, may substantially raise the respiratory syndrome coronavirus 2 (SARS-CoV-2) mortality [29]. In this review, we summarize the recent research on diarylureas with antimicrobial and antiviral activities and other properties related to pandemics. The literature data reported herein make these molecules attractive in this area.

## 2. Diarylureas with Antimicrobial Activity

Antimicrobials are small molecules that can inhibit or kill bacteria. However, some bacteria can grow and survive despite antimicrobial pressures, a characteristic known as antimicrobial resistance. In clinical settings, resistant bacterial infections decrease the achievable treatment options and increase morbidity and mortality, compared with those caused by susceptible microbes [30]. Moreover, traditional bactericidal antibiotics kill only actively growing bacterial cells, whereas non-growing metabolically inactive cells are tolerant to and therefore “persist” in the presence of legacy antibiotics. Thus, the discovery of new antibiotics may be envisaged. Triclocarban (TCC, Table 1 and Table 2) is a diarylurea used as an antimicrobial agent in feminine care products, such as bar soaps, deodorants, detergents, and other disinfectants [31]. Besides its antimicrobial activity, TCC has been acknowledged as an endocrine disruptor, i.e., “an exogenous substance or mixture that alters function(s) of the endocrine system and consequently causes side effects in an intact organism, or its progeny, or (sub)populations” (World Health Organization 2013) resulting in hormonal effects [32]. Moreover, TCC demonstrated anti-inflammatory effects by inhibiting soluble epoxide hydrolase (sEH) in a lipopolysaccharide (LPS)-challenged murine model [33]. It has been demonstrated that TCC alter cardiac functions [34] and is associated to potential adverse birth outcomes in neonates, in particular decreased gestational age at birth [35]. The biodegradation of TCC is very slow as a result of its three chlorine atoms; thus, it can persist in the environment for years and it accumulates in aquatic habitats [36]. For these reasons, since 2016, the Food and Drug Administration (FDA) has banned its use in consumer products [37]. The mechanism of action against bacteria is still unknown. Unlike other antibacterial compounds, triclocarban does not interfere with the membrane. Therefore, it may act as an inhibitor of lipoprotein synthesis, by mimicking the natural substrate of enoyl-acyl-carrier protein reductase (ENR) enzyme. ENR is a highly conserved enzyme of lipid biosynthetic pathways in bacteria, notably gram-negative, gram-positive, and mycobacterial species [38]. Macsics et al. (2020) recently studied the TCC mechanism of action. Protein demethylmenaquinone methyltranferase (MenG) was proposed as the molecular target of TCC, which inhibits its biosynthesis finally leading to inhibition of the *S. aureus* menaquinone metabolism [39].

### 2.1. Diarylureas with Antiparasitic Activity

Diarylureas with antiparasitic activity are summarized in Table 1. TCC and several analogues have been studied in schistosomiasis. This disease, also called bilharzia, is one of the most dramatic parasitic diseases in tropical countries and remains a serious public health problem in the tropics and subtropics, affecting one billion people, with 250 million infected in 74 countries [40]. It is considered one of the most widespread infectious diseases among the WHO-prioritized 17 neglected tropical diseases (NTDs) [41]. Schistosomiasis is caused by the trematode worms of the genus *Schistosoma* (Platyhelminthes Trematoda). The clinically most relevant species are *Schistosoma japonicum*, *S. haematobium*, and *S. mansoni* [42]. *S. japonicum* is responsible for intestinal and hepatosplenic schistosomiasis in China, the Philippines, and Indonesia; *S. haematobium* determines urogenital schistosomiasis in Africa and in some countries of the Arabian Peninsula (it has also recently emerged on the French island of Corsica); *S. mansoni* causes intestinal and hepatic disease in Africa, the Arabian Peninsula, and Latin America [43]. Praziquantel (PZQ) discovered in the 1970s, is the only drug available for the treatment of schistosomiasis [44]; no schistosomiasis vaccines have been accepted for public use yet [45]. Despite many benefits of PZQ, most notably its high efficacy and excellent tolerability, the drug has important disadvantages, above all its inefficacy against juvenile schistosomes. Furthermore, the increasing administration of PZQ to millions of people annually results in high drug pressure, and thus drug-resistant parasites are likely to evolve. The Medicines for Malaria Venture (MMV) Malaria Box is a collection of over 400 compounds including families of structures identified in phenotypic screens of pharmaceutical and academic libraries against the *Plasmodium falciparum* malaria parasite [46]. The antischistosomal properties of some of these compounds with confirmed in vitro activity against *P. falciparum* were studied. Diarylurea MMV665852, a structural analog of TCC, which showed activity against *P. falciparum* 3D7, was also evaluated against *S. mansoni* in vitro on newly transformed schistosomula (NTS) and adult *S. mansoni* worms and showed IC_50_ values of 4.7 and 0.8 µM, respectively. All the compounds were then tested in vivo using the chronic *S. mansoni* mouse model. The compound MMV665852 showed the highest in vivo activity, given that treatment of *S. mansoni* infected mice with a single oral 400 mg/kg dose of MMV665852 reduced worm burden by 52.5% [47]. Then, a search for structures similar to that of MMV665852, using a Tanimoto-Rogers similarity coefficient of 0.85 as the cutoff, identified 46 compounds, 13 of which were diarylureas. All compounds were first tested in vitro on NTS and adult *S. mansoni* worms. MMV665852, TCC and **1** showed IC_50_ = 4.7, 0.07, and 1.3 µM, respectively against NTS; TCC and **1** showed IC_90_ of 0.3 and 2.8 µM, respectively, against NTS. TCC, compounds **1** and **2**, tested at 33.3 µM, showed high in vitro activity against adult *S. mansoni*, showing IC_50_ values of 0.4, 0.7 and 0.2 µM, and IC_90_ of 1.4, 2.2 and 0.8 µM, respectively, after a 72-h incubation period. Interestingly, compounds **3** and **4** were found to be schistosomicidal against the adult *S. mansoni* 72 h post incubation, with calculated IC_50_ values are of 3.6 and 7.0 µM, respectively [48]. In another work, in order to enlarge the structure-activity relationships for compounds analogues of MMV665852, a series of diarylureas was studied in vitro against the juvenile *S. japonicum*. Compounds **5** and **6**, tested at 32 μM, killed the juvenile worms within 72 h, and exhibited a higher activity than the positive control MMV665852 (IC_50_ = 2.5 and 2.8 μM versus 4.4 μM of MMV665852). IC_50_ values against the adult worms were lower than those for juvenile worms (IC_50_ = 1.5 and 2.2 μM versus 2.2 μM of MMV665852) [49]. However, compound **6** had been described previously in the literature, as an insecticide known to be highly toxic to fish and invertebrates, named flucofuron [50,51]. Recent studies also address it as a toxicant [52,53]. Wu et al. studied twenty diarylureas and found that compound **7** had antischistosomal activity against *S. mansoni* higher than that of MMV66558, by killing the NTS at a concentration of 10 µM. This compound killed both NTS and adult *S. mansoni* with IC_50_ values of 0.15 and 0.19 µM, respectively. In this paper, studies regarding the relationships existing between lipophilicity and metabolic stability were also carried out. The authors found that there were no clear relationships between lipophilicity and metabolic stability. Compound **7** had the best in vivo antischistosomal activity, with worm burden reduction value of 40% following administration of single 100 mg/kg oral doses [19]. In the last years, protein kinase inhibitors were used as starting points for drug discovery in studies on helminth growth and development. Sorafenib and its structurally related analogue regorafenib were tested in vitro against the larval and adult stage of *S. mansoni* and they were lethal to NTS within 24 h (IC_50_ = 4.1 and 1.1 µM, respectively, for sorafenib; IC_50_ = 6.9 and 1.0 µM, respectively for regorafenib). Sorafenib caused the death of all adults, at 33.3 μM, within 24 h [54].

### 2.2. Diarylureas with Antibacterial and Antifungal Activity

In Table 2, antibacterial and antifungal diarylureas are described. The work by Pujol et al. [55] was devoted to diarylureas designed to overcome the toxicological effect of TCC due to the three chlorine atoms. One or more chlorine atoms of TCC were reduced and/or replaced by pentafluorosulfanyl groups, bioisosteres of the trifluoromethyl groups. Some of the newly synthesized compounds exhibited high potency, broad spectrum of antimicrobial activity against Gram-positive bacteria, and high selectivity index, while displaying a lower spontaneous mutation frequency than TCC. Preliminary experiments suggested a bactericidal mode of action for this family of ureas. Moreover, some of the new molecules removed preexisting *S. aureus* biofilms, which is important in food industry as well as in hospital settings and displayed a lower spontaneous mutation frequency in *S. aureus* than TCC. Compound **8** emerged as the most promising compound showing the highest potency against both *S. aureus* ATCC 12600 and methicillin-resistant *S. aureus* MRSA (MIC_50_ = 0.05 µg/mL versus MIC_50_ = 0.5 µg/mL of TCC), a broader spectrum of activity, and a higher selectivity index [55]. Le et al. (2020) has recently studied 72 compounds deriving from sorafenib as antibacterials. Compound PK150 showed anti-bacterial activity against several pathogenic strains of *S. aureus* at sub-micromolar concentrations. MIC value against methicillin sensitive (MSSA) *S. aureus* NCTC 8325 was 0.3 µM for PK150 and 3 µM for both sorafenib and regorafenib (MIC = 1 and 3 µM for vancomycin and linezolid, respectively). It exhibited a 10-fold enhanced anti-MRSA activity, lack of resistance development under laboratory conditions, killing of persisters, elimination of established biofilms, and in vivo efficacy in a mouse model. Chemical proteomic studies did not reveal a known kinase as target, but interference with menaquinone biosynthesis and dysregulation of protein secretion as putative target mechanisms [56]. Macsics et al. (2020) studied the mechanism of PK150. As TCC, it is an inhibitor of MenG biosynthesis, but unlike TCC, it is not affected by environmentally acquired TCC resistance as it causes over-activation of SpsB, the bacterial signal peptidase I enzyme from *S. aureus* [39]. The two anticancer agents sorafenib and regorafenib were also investigated as antibacterials and proposed for further studies in this field [57]. Chang et al. (2016) studied antibacterial and antiproliferative activity of sorafenib. The authors compared anti-*Staphylococcus* activity (MIC, Minimum Inhibitory Concentration) versus antiproliferative activity (IC_50_) of test agents against HEK-293 human embryonic kidney, K-562 human erythromyeloblastoid leukaemia, HT-29 human colon adenocarcinoma cell lines. It showed antibacterial activity against *S. aureus* NCTC 8325, with MIC_90_ (i.e., concentration that inhibits 90% of bacteria strains tested) = 4 mg/L. However, the authors concluded that it lacked selectivity, having a low selectivity ratio (IC_50_ for human cells/MIC for *S. aureus*) of 0.7–0.8, thus assessing it is not a feasible candidate for treatment of MRSA infection. Then, the authors studied other diarylureas and found that compounds **6** was highly potent against *S. aureus* (NCTC 8325 and ATCC 12598) and *S. epidermidis* (ATCC 12228 and ATCC 35984) strains, with MIC = 0.25 mg/L. It was classified as bactericidal. The selectivity ratio for this compound was higher than sorafenib, ranging between 4 and 19 [58]. In 2004, Francisco et al. [59] synthesized and studied a series of phenyl thiazolyl urea as new inhibitors of the bacterial cell-wall biosynthesis. Indeed, the peptidoglycan biosynthesis requires 10 synthetic transformations and, correspondingly, 10 specific enzymes, including MurA and MurB. Phenyl thiazolyl urea **9** demonstrated a good activity against MurA and MurB and gram-positive bacteria including MRSA, vancomycin resistant Enterococcus (VRE), and penicillin-resistant Streptococcus pneumoniae (PRSP), with MIC values ranging between 0.5 and 8.0 µg/mL. However, when tested in the presence of 4% bovine serum albumin, their MICs increased significantly [59]. The importance of compound **9** is underlined by its use as a positive control for studies on MurB [60]. Hassan et al. (2014) reported a study on diarylureas with activity against bacteria and fungi. Compound **10** possessed antimicrobial activity against the tested Gram-positive bacterium *Bacillus subtilis* (NCTC-10400) and Gram negative bacteria *Pseudomonas aeruginosa* (ATCC 10145) and *Escherichia coli* (ATCC 23282) showing mean values of inhibition zones (in mm) of 14.0 versus 34.0, 32.0 and 30.0 of erythromycin, respectively. The compound showed weak antifungal activity [61]. Diarylurea PQ401 is a small molecule previously described as inhibitor of the insulin like growth factor I receptor (IGF-1R) signaling and then studied in breast cancer and osteosarcoma [15]. Recently, it has been proposed as a lead candidate for repurposing as a membrane-active antimicrobial agent because it is able to kill both growing and non-growing antibiotic-tolerant MRSA by lipid bilayer disruption. When tested against a panel of antibiotic-resistant *S. aureus* strains (MW2, ATCC 33591, JE2, BF1‒5, BF7,8, BF10,11), including MRSA clinical isolates and a vancomycin-resistant *S. aureus* (VRSA; strain VRS1), it showed a MIC value of 4 µg/mL. PQ401 was demonstrated to be active against MRSA (MW2, ATCC 33591, JE2) and VRS1 strains showing a minimum bactericidal concentration (MBC) of 4 g/mL. Unlike other well-studied membrane-disrupting cationic antimicrobial low-molecular-weight antimicrobials, maximum membrane activity was shown by PQ401 in its neutral form rather than its cationic form. PQ401 also showed efficacy in both the *Caenorhabditis elegans* and *Galleria mellonella* models of MRSA infection [62]. A series of the diarylureas was synthesized and screened for antimicrobial activity against Gram positive and negative bacteria and fungi. Compounds **11** and **12** showed activity against *Proteus mirabilis* ATCC 19181 comparable to that of standard ciprofloxacin (zone of inhibition in 23 and 24 mm, respectively, at a concentration of 200 µg/mL compared to 30 mm of ciprofloxacin) [63]. In the work by Gezegen et al. (2017), a series of diarylureas was studied as antimicrobial and antiproliferative agents. Antimicrobial activity studies were carried out against Gram-positive and Gram-negative bacteria and yeasts, by using piperacillin/tazobactam (P/T = 8/1) and fluconazole as positive controls. The most interesting compounds were **13** and **14**. They were both more active than reference against *Shigella boydii* ATCC 9905 and *Enterococcus faecalis* ATCC 29212 (MIC = 31.3 µg/mL versus 62.5 µg/mL), a clinically significant pathogen implicated in different types of infections [64], and against *Bacillus cereus* ATCC 10987 (MIC = 31.3 µg/mL versus 125 µg/mL), while they showed the same activity of the reference against *Klebsiella pneumoniae* ATCC 10031 (MIC = 31.3 µg/mL) [65]. Diarylureas **15–17**, bearing an aminoguanidine group that is a common moiety in medicinal chemistry [66], were active against MRSA NRS123 showing MIC values of 10 µg/mL (for compounds **15** and **17**) and 8 µg/mL (for compound **16**) [67]. Compound **16** was chosen as the lead compound for further studies on a series of diarylureas bearing an alkoxy side chain in lieu of the *n*-butyl moiety. The substitution with an isopentyloxy or cycloheptyloxy group gave compounds **18** and **19**, which showed activity in vitro against MRSA (MIC values between 2 and 4 µg/mL versus vancomycin ranging from 0.5 to 1 µg/mL). Compound **19** was chosen for further in vivo studies using a *C. elegans* animal model. The antibacterial activity was confirmed as **19**, at 10 µg/mL, reduced the burden of MRSA USA400 by more than 50% in infected worms, which is a result better than that observed for vancomycin (at the same concentration it reduces the bacterial burden by 25%). Compound **19** also showed better pharmacokinetics, showing enhanced stability to hepatic metabolism, it was also demonstrated to be suitable for intravenous or topic administration (for treatment MRSA skin infections) [67]. In a sequent work, the investigation of action mechanism of a series diphenylureas revealed that they exert their antibacterial effect by interfering with bacterial cell wall synthesis. Interestingly, both compounds **18** and **19** are able of re-sensitizing VRSA to the effect of vancomycin. Furthermore, compound **18** can penetrate staphylococcal biofilms (*S. aureus* and *S. epidermidis*) to reduce the burden of bacteria present within the biofilm, although at high concentrations [68]. Upadhayaya et al. (2009) studied several quinoline derivatives as antimycobacterials. Tuberculosis (TB) is an old human disease and represent a major threat for mankind especially because of the emergence of resistance strain of *Mycobacterium tuberculosis* against antibiotics. Compounds **20** and **21** inhibited *M. tuberculosis* H37Rv up to 98% and 94%, respectively, at a concentration of 6.25 µg/mL. They showed MIC values of 6.25 µg/mL and 3.125 µg/mL, respectively [69]. Compounds **22** and **23** showed MIC values of 6.0 and 5.2 µg/mL, respectively, against *M. tuberculosis* pathogenic strain H37Rv and 2.0 and 1.0, respectively, against *M. tuberculosis* nonpathogenic strain mc^2^6030. They also showed selective inhibition of mycolic acid biosynthesis. At the same time, these molecules also executed their potent immunomodulatory activity by up-regulation of the pro-inflammatory cytokines IFN-g and IL-12 and down-regulation of IL-10 [70]. Compound **24** was studied for antifungal activity, containing 1,2,4-triazole, showed good antifungal activity against *Phomopsis* species. These are known as *Phomopsis* cane and leaf spot (*P. viticola*) causes economic losses to the vine grape production in the USA and Europe, while *P. obscurans* is responsible of *Phomopsis* leaf blight and fruit rot of strawberry. At 30 μM, compound **24** inhibited the growth of *P. obscurans* and *P. viticola* by 80% and 100%, respectively, after 120 h exposure, showing an activity similar to that of the positive control Captan, a well-known multisite inhibitor fungicide with no systemic activity, used as a commercial protectant fungicide to prevent anthracnose diseases in fruits and ornamentals [71].

## 3. Diarylureas with Antiviral Activity

Today, viruses represent the most common cause of infections in the world; therefore, research is being conducted to identify new safe and effective antivirals [72]. Among the several virus families, the *Flaviviridae* one has attracted the attention of some research groups working on the diarylureas field. It includes Dengue virus (DENV), West Nile virus (WNV), and Yellow fever virus (YFV), Zika virus (ZIKV), many of which cause serious human and animal infections. Micewicz et al. (2018) described the identification of potent inhibitors of Zika virus infection, which is a mosquito-borne RNA virus, with picomolar activity in vitro. Compounds ASN 07115854, ASN 07115873, ASN 07115881, and ASN 07115927 (Table 3) were active in the low nanomolar or picomolar range (IC_50_ = 160.3 nM, 189.2 pM, 317.7 pM and 26.9 nM, respectively) as assessed by using a viral plaque-forming assay and the A549 human lung carcinoma cell line, described as highly permissive to Zika infection [73]. In a successive paper by the same research group, a new library of diarylureas was screened using the same assay. Several compounds in the library showed high inhibitory activity against Zika virus with IC_50_ values in the picomolar range. The most interesting compound was **25** which exhibited an excellent IC_50_ = 85.1 pM [74]. Recently, several diarylureas have been studied for the treatment of DENV. New compounds binding the flavivirus NS5 AdoMet-dependent mRNA methyltransferase (MTase) domain, a main component of DENV replication complex, were proposed using a fragment-based drug design. Compounds **26** and **27** were the most interesting compounds, as they showed high inhibitory effect in both DENV N7-MTase assay (IC_50_ = 91 µM and 110 µM, respectively) and in WNV 2′-*O*-MTase assay (IC_50_ = 51 µM and 71 µM, respectively) [75].

## 4. Diarylureas as Antimalarial Agents

Diarylurea MMV665852, the structural analog of TCC mentioned above, was also studied for antimalarial activity and showed an EC_50_ = 1160 nM against *P. falciparum* 3D7 (Table 3). Plasmepsins are aspartic proteases that malarial parasites use to digest hemoglobin during the intraerythrocytic stage. Diphenylurea derivative WR268961, obtained from the Walter Reed chemical inventory, was found to inhibit recombinant plasmepsins, which possess kinetic similarity to the native enzymes, with a *K*_i_ value between 1 and 6 µM. It inhibited the growth of *P. falciparum* strains W2 and D6, with IC_50_ ranging from 0.03 to 0.16 µg/mL and showed low toxicity to mammalian cells. Some derivatives of WR268961 in which the amidine moiety was replaced by a sulfonic acidic group, retained plasmepsin inhibitory activity but were poor inhibitors of *P. falciparum* growth in vitro (figures not shown) [76]. Zhang et al. 2010 studied a large library of diarylureas for antimalarial activity against chloroquine-sensitive (3D7) and chloroquine-resistant (K1) strains of *P. falciparum*. PQ401, showed the same potency of chloroquine (EC_50_ = 0.053 μM versus 0.047 μM) against the 3D7 cell line in vitro. Compounds **28** and **29** showed higher activity than the parent compound, showing EC_50_ values of 0.32 and 0.031 μM, respectively, against the same cell line. The most interesting compound of the series was **33** which was 3-fold more potent than chloroquine (EC_50_ = 0.016 μM). Compounds **29** and **30** also showed high activity against chloroquine resistant K1 strain (EC_50_ = 0.11 and 0.079 μM, respectively, versus 1,23 μM of chloroquine) [20]. Compound **31** demonstrated EC_50_ values of 0.037 µM and 0.055 µM against in vitro cultured parasite 3D7 (drug-sensitive) and Dd2 (resistant to chloroquine and pyrimethamine–sulfadoxine) *P. falciparum* strains, respectively. It also demonstrated in vivo efficacy in a *Plasmodium berghei* antimalarial mouse model, with >50% survival at day 31 post-treatment after subcutaneous administration at 256 mg/kg [77].

## 5. Diarylureas as Anti-Inflammatory Agents and Antiulcer Agents

The inhibition of Δ5-desaturase by small molecules may attenuate inflammatory responses, potentially creating next-generation anti-inflammatory agents. Diarylurea CP-214339 (Table 4) acts as inhibitor of a Δ5-desaturase in rodents (IC_50_ = 0.13 µM), thus decreasing arachidonic acid synthesis [78,79]. Several diarylureas, in which one aryl moiety is represented by a substituted dihydropyrazole, were studied for their anti-inflammatory and antimicrobial activities. In vitro anti-inflammatory activity was tested against the pro-inflammatory cytokines (TNF-α and IL-6) by TNF-α and IL-6 inhibition assay. Compounds **32–34** exhibited anti-inflammatory activity, showing TNF-α (85%, 58% and 61%, respectively) and IL-6 (93%, 75% and 80%, respectively) inhibitory activity as compared to the standard dexamethasone but at a higher concentration (10 µM). They showed low or no antimicrobial activity [80]. Rakesh et al. (2017) studied a series of disubstituted ureas for their in vitro H^+^/K^+^ ATPase (proton pump) and anti-inflammatory activity. Diarylurea **35** behaved as an antiulcer, exhibiting higher activity against proton pump than omeprazole (IC_50_ = 18.4 µg/mL versus 38.2 µg/mL), while compound **36** displayed higher anti-inflammatory activity than indomethacin (IC_50_ = 20.3 µg/mL versus 44.8 µg/mL) [81].

## 6. Diarylureas as Integrine Antagonists

The inhibitors of integrin α4β1, also known as VLA-4 (Very Late Antigen-4), might serve as useful agents in the treatment of asthma, rheumatoid arthritis, inflammatory bowel disease, multiple sclerosis, Alzheimer’s disease (AD), and stroke [82]. In interstitial pneumonia (IP), integrin α4β1 expression level is known to be up regulated by inflammatory cytokines [83]. The major cause of death of COVID-19 patients is characterized by respiratory failure due to IP [84]. Compounds **37** and **38** (Table 4) were identified as potent antagonists of VLA-4 showing p*K*_i_ values of 7.2 and 9.1, respectively [85]. Witherington et al. (2020) studied new diarylureas and found compounds **39** and **40** displaying excellent inhibitory potency (p*K*_i_ = 8.7 and 9.3, respectively) [86].

## 7. Diarylureas as Allosteric Modulators of Cannabinoid Receptor 1

The activation of cannabinoid receptor 1 (CB_1_) is essential for the development of an effective innate immune response during bacterial infection [87]. Diarylurea PSNCBAM-1 is an allosteric modulator of CB_1_ and antagonized G protein coupling [88]. Starting from this lead compound, several analogues have been studied. Compounds **41** and **42** (Table 4), bearing a pyrimidine ring, were shown act as allosteric modulators of CB_1_ while showing an antagonism of G-protein coupling activity. They ascertained extracellular signal-regulated kinases ERK1/2 phosphorylation mediated via β-arrestin [89]. In a recent work, several restricted derivatives of PSNCBAM-1 were studied. Compounds LDK1317 and LDK1321 exhibited binding affinity (*K*_B_) and binding cooperativity factor (α) of *K*_B_ = 110 nM, α = 2.3 (for LDK1317) and *K*_B_ = 85 nM, α = 5.9 (for LDK1321) and had computationally predicted drug-like properties and better solubility than the parent compound [90].

## 8. Diarylureas as Antiplatelet Agents

Adenosine-5′-diphosphate (ADP) plays an essential role in platelet aggregation and thrombus formation. Two G-protein coupled P2 purinergic ADP receptors, P2Y_1_ and P2Y_12_, are both required for full ADP-induced platelet aggregation. In recent years, the P2Y_1_ receptor has emerged as an attractive target for anti-thrombotic therapies from preclinical antithrombotic studies and haemorragic models. P2Y_12_ receptor antagonists are predicted to blunt platelet activation and they may exert beneficial actions on other cell types affected by COVID-19 [91]. Diarylurea BPTU showed good binding affinity with P2Y_1_ receptor (*K*_i_ = 6 nM) and moderate antiplatelet activity in the ADP-induced platelet aggregation assay in vitro. The pharmacokinetic profile of BPTU revealed that this compound had a moderate half-life (t_1/2_ = 1.43 h) and bioavailability (F = 18%) when orally dosed at 30 mg/kg in rats [92]. From the crystal structures of BPTU binding with P2Y_1_ receptor it emerged that BPTU bound to P2Y_1_ receptor on the lipid interface of the transmembrane domain, thus indicating BPTU as an allosteric modulator of P2Y_1_ receptor [93]. Starting from BPTU as a lead compound, several other analogues were then synthesized. Wang et al. (2013) demonstrated that compounds **43** and **44** (Table 4) showed significantly higher aqueous solubility while maintaining antiplatelet activity similar to that of BPTU. Compound 43 was efficacious and showed a dose-dependent reduction of thrombosis weight with an EC_50_ of 2 µM in the rabbit electrolytic-mediated carotid arterial thrombosis (ECAT) model and had a moderate prolongation of bleeding time in rats similar to that of compound BPTU. However, it showed toxicity in the rabbit [94]. A novel series of 2-(phenoxyaryl)-3-urea derivatives were designed, synthesized, and biologically evaluated for their anti-thrombotic activity and compared to BPTU. Compound **45** demonstrated a good P2Y_1_ receptor antagonistic potency in vitro (IC_50_ = 0.21 µM versus 0.28 µM of BPTU). In a study on antiplatelet aggregation, compounds **46** and **47** exhibited good antiplatelet activity (IC_50_ = 12.52 and 7.19 µM, respectively). The possible binding modes of compounds with P2Y1 receptor were also explored by molecular docking simulation. The docking studies demonstrated that compound **45** interacted well with Phe119 through hydrophobic interaction and modestly improved the P2Y1 receptor antagonistic activity, making it justifiable for further investigation [95]. Qiao et al. (2013) reported new conformationally constrained ortho-anilino diarylureas. Compound **48** acts as P2Y_1_ antagonist, showing a *K*_i_ value of 4.3 nM in the primary membrane binding assay and improved in vitro functional potency in the fluorescent imaging plate reader (FLIPR) P2Y_1_ assay (IC_50_ = 2.5 nM). It showed a strong antithrombotic effect with mild bleeding liability in the rat thrombosis and hemostasis models, showing IC_50_ = 4.9 µM in the ADP-induced platelet aggregation (PA) assay [96]. In a successive study, the benzothiazole-containing diarylurea **52** was demonstrated to be a potent P2Y_1_ antagonist and orally bioavailable with a suitable PK profile in preclinical species. It showed a good binding affinity (*K*_i_ = 7.9 nM) and improved in vitro functional potency in the FLIPR P2Y_1_ assay (IC_50_ = 0.12 nM). It also showed a strong antithrombotic effect in the ADP-induced PA assay (IC_50_ = 0.13 µM), better than the previously described **48** and enhanced haemorragic profile compared to P2Y_12_ antagonist clopidogrel in rat efficacy/bleeding models [97]. Then, other diarylureas were studied. Compound **50** was slightly stronger in binding affinity (*K*_i_ = 4.0 nM) but much stronger in FLIPR assay (IC_50_ = 0.22 nM) and was equiactive in the inhibition of platelet aggregation (PA IC_50_ = 0.17 µM). The spiropiperidinyl indoline-containing diarylurea **51**, retained a good in vitro potency (*K*_i_ = 12.9 nM) and was much more active in the in vitro functional assays with FLIPR (IC_50_ = 0.22 nM) and PA (IC_50_ value of 0.41 µM). Compound **51** also showed the highest metabolic stability and lowest clearance (CL) value (thus, high i.v. exposure), small volume of distribution (V_dss_) values, and modest bioavailability [98].

## 9. Diarylureas Acting on Central Nervous System

Several diarylureas were described in the literature for their activities on CNS. Compound **52** (Table 4) was effective as antiepileptic in both maximal electroshock (MES) and subcutaneous pentylenetetrazole (scPTZ) seizure tests, showing ED_50_ value of 28.5 mg/kg against MES induced seizures. Toxicological results revealed that no significant toxicity was detected after administration for 14 days (sub-acute toxicity study) in rats. It also exerted antidepressant activity in forced swim test (FST) and tail suspension test (TST) in mice [99].

## 10. Diarylureas for Neuroinflammation

Neuroinflammation has been connected to neurodegenerative disorders of the brain, such as AD, Parkinson’s disease (PD), and Huntington’s disease (HD). The transient receptor potential vanilloid 1 (TRPV1) plays an essential part in neuroinflammation and has been recently reviewed [100]. Feng et al. (2016) reported a study on diarylureas acting on TRPV1. Compounds **50**–**55** (Table 4) were the most active against TRPV1 (*K*_i_ values for capsaicin antagonism = 0.47, 0.49, 0.56 µM, respectively). Compound **57** also showed potential binding affinity (*K*_i_ = 1.39 μM) at cannabinoid receptor 2 (CB_2_), which is another attractive target for immunoinflammation diseases. Compound **56** was predicted to target the CXC chemokine receptor 2 (CXCR2), which is also a potential target for the treatment of chronic inflammatory diseases. However, the bioassay validation of CXCR2 with this compound was not performed [101].

## 11. Diarylureas as Inhibitors of AChE and BuChE

The genetic predisposition has been suggested to be involved in COVID-19 disease transmission. Genetic polymorphisms in other multiple genes, such as that codifying for the AChE, is relevant to SARS-CoV-2 pathophysiology. Several diarylureas were studied for their action on AChE for the treatment of AD. Compound **57** (Table 4) exhibited intense activity of inhibition on both AChE and butyrylcholinesterase (BuChE), with an IC_50_ = 3.85 and 9.25 µM, respectively; thus, it may act as a dual inhibitor. The selectivity index was 2.40 against AChE and BuChE, respectively [102].

## 12. Summary

This paper reviews the current status and the recent studies of biologically important diarylurea derivatives. The diarylurea moiety may be considered a privileged structure in medicinal chemistry. Besides the widely known antitumor tyrosine kinase inhibitors sorafenib and others, several diarylureas have been studied for a variety of biological activities, including antimicrobial, antiviral, anti-inflammatory and antiplatelet action. The intention of this review is to highlight the profusion of recent biological data that exists around the diarylurea class of compounds, focusing especially on their antimicrobial, antiviral and all the other activities that could be useful for the treatment or prevention of new COVID-19 pandemics, for which there is any evidence of effective treatments. Promising strategies to combat SARS-CoV-2 include the discovery of therapeutic targets, drugs and vaccines/monoclonal antibodies and, considering the emergency and urgency, approved repurposed drugs, which could be important and useful for the therapeutic management of the disease. To date the main therapies used to treat this disease are antiviral drugs, chloroquine/hydroxychloroquine and antibiotics, bronchospasmolitics, beta-blockers, steroidal and nonsteroidal anti-inflammatory drugs and so on). As most of the early-detected bacterial pneumonia can be safely and effectively treated with antibiotics, the broad-spectrum antibiotics are widely used, as well, in COVID-19 patients. Drugs such as remdesivir, chloroquine, ritonavir, tocilizumab, corticosteroids have been repurposed for the treatment of COVID-19, although their clinical effectiveness has not yet been confirmed. Unfortunately, the use of chloroquine and derivatives such as hydroxychloroquine, alone or in combination with other drugs, resulted in cardiac toxicity. Taking into account that COVID-19 is a multi-organ dysfunction, which causes comorbidities or complications and triggers an unregulated systemic inflammatory response, new molecules with multi-target and multi-properties action are need. Recently, new kinases inhibitors have been reported for their implications in modulating the inflammatory response, which is, as well, one of the major issue during the COVID-19. The different biological activities herein described for diarylureas, namely antibacterial, antiviral, anti-inflammatory and anticoagulant, may be very effective in hampering the different steps of SARS-COV-2 diffusion and progression, influencing positively the above mentioned comorbidities. Moreover, the already known anti-bacterial properties make them promising molecules for repositioning diarylureas as antimicrobial agents and interesting scaffolds for the development of multitarget COVID-19 treatments.

## Figures and Tables

**Table 1:** Diarylureas as antiparasitic agents.

Structure	Compd	Activity	Ref
*Antiparasitic agents*			
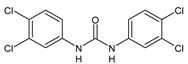	**MMV665852**	IC_50_ = 4.7 µM (*S. mansoni* NTS) IC_50_ = 0.8 µM (adult *S. mansoni*) IC_50_ = 4.4 µM (juvenile *S. japonicum*) IC_50_ = 2.2 µM (adult *S. japonicum*)	[47] [49]
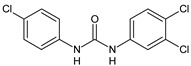	**Triclocarban (TCC)**	IC_50_ = 0.07 µM (*S. mansoni* NTS) IC_50_ = 0.4 µM (adult *S. mansoni*)	[48]
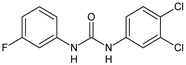	**1**	IC_50_ = 1.3 µM (*S. mansoni* NTS) IC_50_ = 0.7 µM (adult *S. mansoni*)	[48]
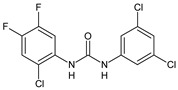	**2**	IC_50_ = 0.2 µM (adult *S. mansoni*)	[48]
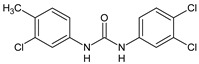	**3**	IC_50_ = 3.6 µM (adult *S. mansoni*)	[48]
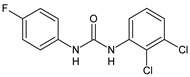	**4**	IC_50_ = 7.0 µM (adult *S. mansoni*)	[48]
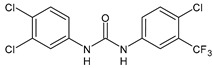	**5**	IC_50_ = 2.5 µM (juvenile *S. japonicum*) IC_50_ = 1.5 µM (adult *S. japonicum*)	[49]
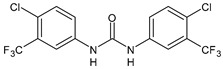	**6 (Flucofuron)**	IC_50_ = 2.8 µM (juvenile *S. japonicum*) IC_50_ = 1.5 µM (adult *S. japonicum*)	[49]
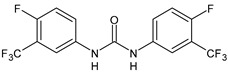	**7**	IC_50_ = 0.15 µM (*S. mansoni* NTS) IC_50_ = 0.19 µM (adult *S. mansoni*)	[19]
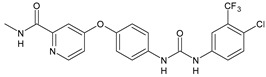	**Sorafenib** **(BAY-43-9006)**	IC_50_ = 4.1 µM (*S. mansoni* NTS) IC_50_ = 1.1 µM (adult *S. mansoni*)	[54]
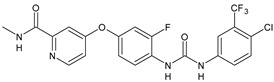	**Regorafenib (BAY-73-4506)**	IC_50_ = 6.9 µM (*S. mansoni* NTS) IC_50_ = 1.0 µM (adult *S. mansoni*)	[54]

**Table 2:** Diarylureas as antibacterials and antifungals.

Structure	Compd	Activity	Ref
*Antibacterial activity*			
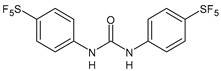	**8**	MIC_50_ = 0.05 µg/mL (*S. aureus* ATCC 12600 and MRSA)	[55]
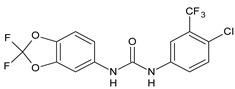	**PK150**	MIC = 0.3 µM (*S. aureus* NCTC 8325)	[56]
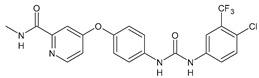	**Sorafenib** **(BAY-43-9006)**	MIC = 3 µM (*S. aureus* NCTC 8325)	[56]
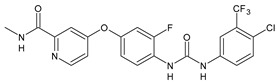	**Regorafenib** **(BAY-73-4506)**	MIC = 3 µM (*S. aureus* NCTC 8325)	[56]
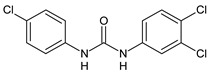	**Triclocarban** **(TCC)**	MIC_50_ = 0.5 µg/mL (*S. aureus*)	[55]
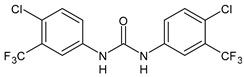	**6** **(Flucofuron)**	MIC = 0.25 mg/L (*S. aureus* NCTC 8325; ATCC 12598 and *S. epidermidis* ATCC 12228; 35984)	[58]
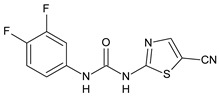	**9**	MIC = 0.5‒8.0 µg/mL (different gram-positive bacteria)	[59]
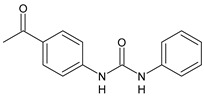	**10**	Inhibition zones = 14.0 mm Gram (+) *B. subtilis* NCTC-10400 and Gram (‒) *P. aeruginosa* ATCC 10145 and *E. coli* ATCC 23282	[61]
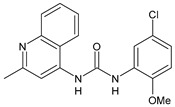	**PQ401**	MIC = 4 µg/mL (different *S. aureus*) MBC = 4 g/mL (*S. aureus* VRS1 strains)	[62]
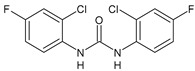	**11**	Inhibition zone = 23 mm, at a concentration of 200 µg/mL (*P. mirabilis* ATCC 19181)	[63]
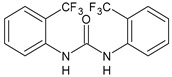	**12**	Inhibition zone = 24 mm, at a concentration of 200 µg/mL (*P. mirabilis* ATCC 19181)	[63]
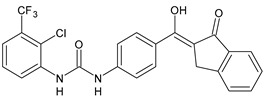	**13**	MIC = 31.3 µg/mL (*S. boydii* ATCC 9905; *E. faecalis* ATCC 29212; *B. cereus* ATCC 10987; *K. pneumoniae* ATCC 10031)	[65]
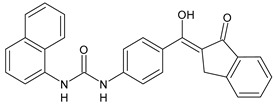	**14**	MIC = 31.3 µg/mL (*S. boydii* ATCC 9905; *E. faecalis* ATCC 29212; *B. cereus* ATCC 10987; *K. pneumoniae* ATCC 10031)	[65]
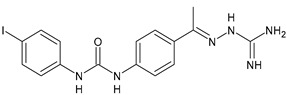	**15**	MIC = 10 µg/mL (MRSA NRS123)	[67]
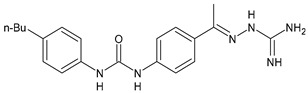	**16**	MIC = 8 µg/mL (MRSA NRS123)	[67]
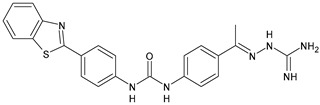	**17**	MIC = 10 µg/mL (MRSA NRS123)	[67]
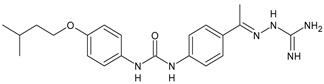	**18**	MIC = 2‒4 µg/mL (various MRSA)	[67,68]
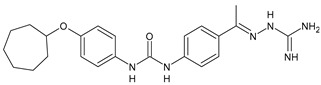	**19**	MIC = 2‒4 µg/mL (various MRSA)	[68]
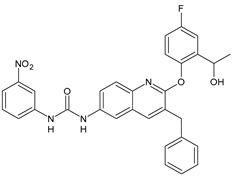	**20**	MIC = 6.25 µg/mL (*M. tuberculosis* H37Rv)	[69]
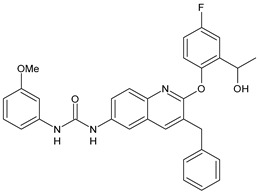	**21**	MIC = 3.125 µg/mL (*M. tuberculosis* H37Rv)	[69]
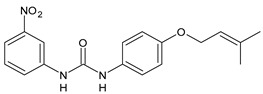	**22**	MIC = 6.0 µg/mL (*M. tuberculosis* pathogenic strain H37Rv) MIC = 2.0 µg/mL (*M. tuberculosis* nonpathogenic strain mc^2^6030)	[70]
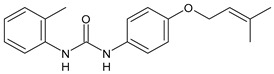	**23**	MIC = 5.2 µg/mL (*M. tuberculosis* pathogenic strain H37Rv) MIC = 1.0 µg/mL (*M. tuberculosis* nonpathogenic strain mc^2^6030)	[70]
*Antifungal activity*			
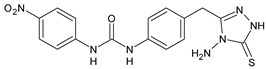	**24**	Inhibition, at 30 μM, of the growth of *P. obscurans* by 80% and *P. viticola* and 100%, after 120 h exposure	[71]

**Table 3:** Diarylureas as antiviral and antimalarial agents.

Structure	Compd		Ref
*Antiviral agents* *Inhibitors of Zika virus infection*			
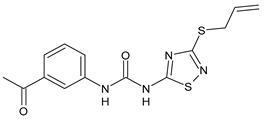	ASN 07115851	IC_50_ = 160.3 nM (by viral plaque assay and A549 human lung carcinoma cell line)	[73]
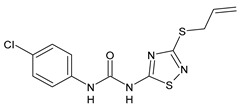	ASN 07115873	IC_50_ = 189.2 pM (by viral plaque assay and A549 human lung carcinoma cell line)	[73]
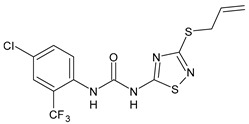	ASN 07115881	IC_50_ = 317.7 pM (by viral plaque assay and A549 human lung carcinoma cell line)	[73]
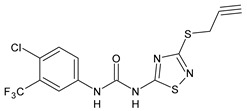	ASN 07115927	IC_50_ = 26.9 nM (by viral plaque assay and A549 human lung carcinoma cell line)	[73]
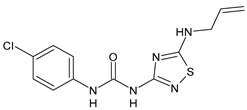	**25**	IC_50_ = 85.1 pM (by viral plaque assay and A549 human lung carcinoma cell line)	[74]
*DENV*			
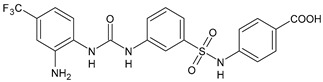	**26**	IC_50_ = 91 µM (DENV N7-MTase assay) IC_50_ = 51 µM (WNV 2′-O-MTase assay)	[75]
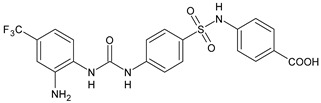	**27**	IC_50_ = 110 µM (DENV N7-MTase assay) IC_50_ = 71 µM (WNV 2′-O-MTase assay)	[75]
*Antimalarial agents*			
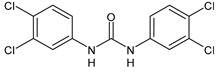	MMV665852	EC_50_ = 1160 nM (*P. falciparum*)	[47]
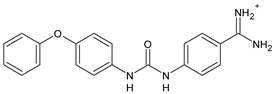	WR268961	IC_50_ = 0.03‒0.16 µg/mL (*P. falciparum* W2 and D6)	[76]
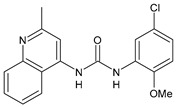	PQ401	EC_50_ = 0.053 μM (*P. falciparum* 3D7 and K1)	[20]
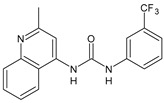	**28**	EC_50_ = 0.32 μM (*P. falciparum* 3D7)	[20]
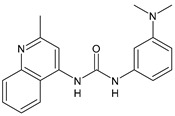	**29**	EC_50_ = 0.031 μM (*P. falciparum* 3D7) EC_50_ = 0.11 μM (*P. falciparum* K1)	[20]
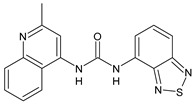	**30**	EC_50_ = 0.016 μM) (*P. falciparum* 3D7) EC_50_ = 0.079 μM (*P. falciparum* K1)	[20]
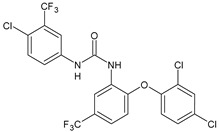	**31**	EC_50_ = 0.037 µM (*P. falciparum* 3D7) EC_50_ = 0.055 µM (*P. falciparum* Dd2)	[77]

**Table 4:** Diarylureas with various biological activities.

Structure	Compd		Ref
*Anti-inflammatory and antiulcer agents*			
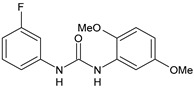	**CP-214339**	IC_50_ = 0.13 µM (Δ5-desaturase inhibitor in rodents)	[78,79]
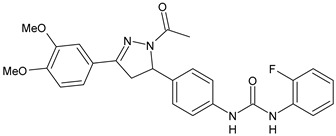	**32**	85% inhibition TNF-α; 93% inhibition IL-6	[80]
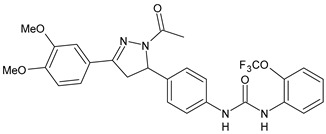	**33**	58% inhibition TNF-α; 75% inhibition IL-6	[80]
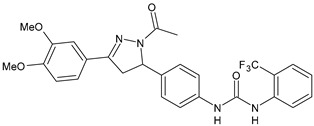	**34**	61% inhibition TNF-α; 80% inhibition IL-6	[80]
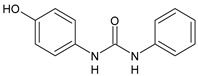	**35**	IC_50_ = 18.4 µg/mL (proton pump inhibition)	[81]
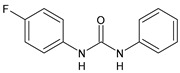	**36**	IC_50_ = 20.3 µg/mL (anti-inflammatory activity in human blood)	[81]
*Integrine antagonists*			
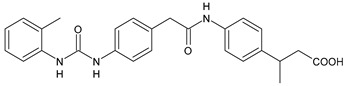	**37**	p*K*_i_ = 7.2 (VLA-4 antagonist)	[85]
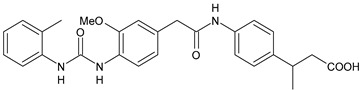	**38**	p*K*_i_ = 9.1 (VLA-4 antagonist)	[85]
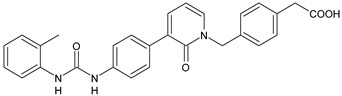	**39**	p*K*_i_ = 8.7 (VLA-4 antagonist)	[86]
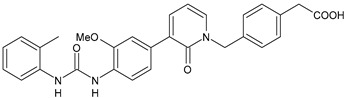	**40**	p*K*_i_ = 9.3 (VLA-4 antagonist)	[86]
*Allosteric Modulators of CB1*			
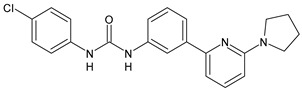	**PSNCBAM-1**	CB_1_ agonist	[88]
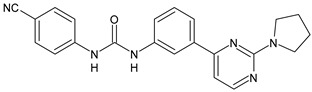	**41**	CB_1_ agonist	[89]
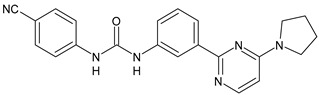	**42**	CB_1_ agonist	[89]
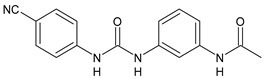	**LDK1317**	CB_1_ agonist	[90]
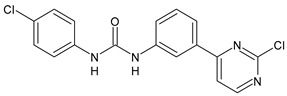	**LDK1321**	CB_1_ agonist	[90]
*Antiplatelet agents*			
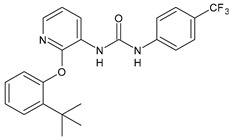	**BPTU**	*K*_i_ = 6 nM (P2Y_1_ receptor) IC_50_ = 0.28 µM (P2Y_1_ receptor)	[92] [95]
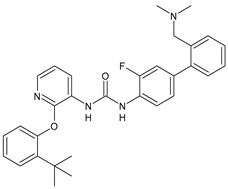	**43**	EC_50_ = 2 µM (ECAT) *K*_i_ = 17 nM (P2Y_1_ receptor)	[94]
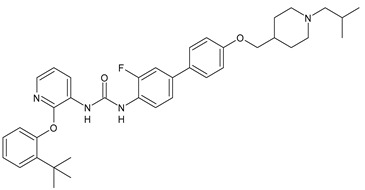	**44**	*K*_i_ = 30 nM (P2Y_1_ receptor)	[94]
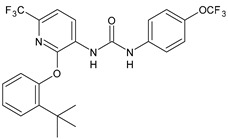	**45**	IC_50_ = 0.21 µM (P2Y_1_ receptor)	[95]
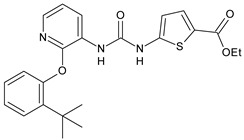	**46**	IC_50_ = 12.52 µM (P2Y_1_ receptor)	[95]
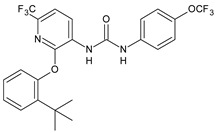	**47**	IC_50_ = 7.19 µM (P2Y_1_ receptor)	[95]
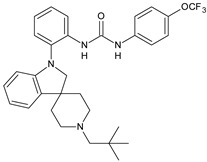	**48**	*K*_i_ = 4.3 nM (P2Y_1_ receptor); IC_50_ = 2,5 nM (P2Y_1_ FLIPR)	[96]
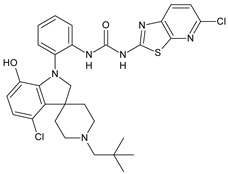	**49**	*K*_i_ = 7.9 nM (P2Y_1_ receptor); IC_50_ = 0.12 nM (P2Y_1_ FLIPR); IC_50_ = 0.13 µM (ADP-induced PA assay)	[97]
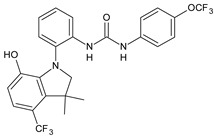	**50**	*K*_i_ = 4.0 nM (P2Y_1_ receptor); IC_50_ = 0.22 nM (P2Y_1_ FLIPR); IC_50_ = 0.17 µM (ADP-induced PA assay)	[98]
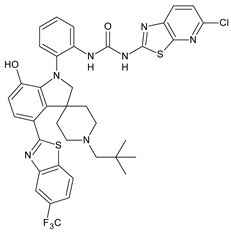	**51**	*K*_i_ = 12.9 nM (P2Y_1_ receptor); IC_50_ = 0.22 nM (P2Y_1_ FLIPR); IC_50_ = 0.41 µM (ADP-induced PA assay)	[98]
*Antiepileptic*			
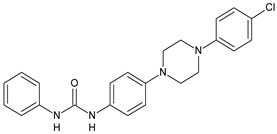	**52**	ED_50_ = 28.5 mg/kg (MES induced seizures)	[99]
*Agents for neuroinflammation*			
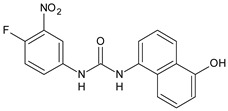	**53**	*K*_i_ = 0.47 µM (TRPV1 capsaicin antagonism)	[101]
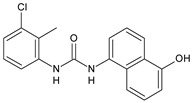	**54**	*K*_i_ = 0.49 µM (TRPV1 capsaicin antagonism); *K*_i_ = 1.39 μM (CB_2_)	[101]
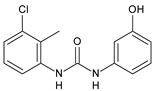	**55**	*K*_i_ = 0.56 µM (TRPV1 capsaicin antagonism)	[101]
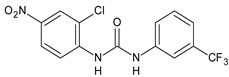	**56**	Binding prediction to CXCR2 (homology model)	[101]
*AChE and BuChE inhibitors*			
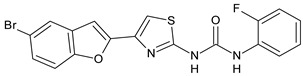	**57**	IC_50_ = 3.85 µM (AChE) IC_50_ = 9.25 µM (BuChE)	[102]

## Data Availability

Data is contained within the article.

## Abbreviations

ACE2	angiotensin converting enzyme 2
AChE	acetylcholinesterase
AD	Alzheimer’s disease
ADP	adenosine-5′-diphosphate
CB_1_	cannabinoid receptor 1
CB_2_	cannabinoid receptor 2
CL	clearance
CNS	Central Nervous System
COVID-19	Coronavirus disease 2019
CXCR2	CXC chemokine receptor 2
DENV	dengue virus
ECAT	electrolytic-mediated carotid arterial thrombosis
ENR	enoyl-acyl-carrier protein reductase
FLIPR	fluorescent imaging plate reader
FST	forced swim test
HD	Huntington’s disease
HFF	human foreskin fibroblast
IGF-1R	insulin like growth factor I receptor
IL-6	interleukin-6
IP	interstitial pneumonia
*K*_B_	binding affinity
LPS	lipopolysaccharide
MenG	demethylmenaquinone methyltranferase
MERS	Swine flu
Ebola	Middle East respiratory syndrome
MES	maximal electroshock
MIC	minimum inhibitory concentration
MMV	Medicines for Malaria Venture
MRSA	methicillin-resistant *S. aureus*
MSSA	methicillin-sensitive *S. aureus*
NTDs	neglected tropical diseases
NTS	newly transformed schistosomula
PGR	plant growth regulator
PA	platelet aggregation
PD	Parkinson’s disease
PZQ	praziquantel
SARS	severe acute respiratory syndrome
SARS-CoV	respiratory syndrome coronavirus
SARS-CoV-2	respiratory syndrome coronavirus 2
scPTZ	subcutaneous pentylenetetrazole
sEH	soluble epoxide hydrolase
Sps	bacterial signal peptidase I enzyme
TB	tuberculosis
TCC	triclocarban
TRPV1	transient receptor potential vanilloid 1
TST	tail suspension test
V_dss_	volume of distribution
VLA-4	very late antigen-4
VRSA	vancomycin-resistant *S. aureus*
WNV	West Nile virus
YFV	Yellow fever virus
ZIKV	Zika virus virus
